# Mouthwashes

**Published:** 1888-04

**Authors:** 


### MOUTHWASHES.

Dr. Miller, in his article upon mouthwashes in this number, calls attention to the fact that the expected results from the late advancement in the knowledge of bacteriological science have not been attained. Practically, we are but little better off than before the investigations of the past few years were made. Yet no one will be so foolish as to declare that they have therefore been in vain, and that the efforts were a waste of time. Definite scientific knowledge must first be obtained, and the laws which dominate conditions must be studied and determined. Their application and the control of conditions must be learned by clinical observation and objective study. When Laplace pointed out that the harmony of our solar system demanded the existence of another planet between the orbits of Mars and Jupiter, the telescopes of the world were at once pointed at the vacant space, with the consequence of the discovery of what was, perhaps, the fragments of a disrupted planet, and the observations were continued until nearly one hundred and fifty Asteroids were added to the solar system. In like manner, now that Dr. Miller has pointed out the probable true factor in dental caries, others will assist in the practical application of these observations.

The observant reader will be impressed by the results of Dr. Miller's late experiments. Some drugs from which he would naturally expect excellent results have proved quite barren, while others are but partially successful. Bichloride of mercury is the only agent which he has found absolutely reliable, and of this most practitioners will stand somewhat in fear because of its toxocological properties. Listerine seems to have been fairly efficacious, and is viewed with favor by Dr. Miller. If the sublimate be an essential, why might not listerine be employed as the base, the dentist making a prescription in which listerine and water should be used to form, say a two per cent. solution of mercuric bichloride ? We have prescribed pure listerine as a mouthwash in cases of extensive dental caries, and with excellent results. In fact, it is the only antiseptic mouthwash that we have felt called upon to prescribe.

We are sure that good results might be attained if intelligent dentists would enter upon a systematic course of experiments with the formulas suggested by Dr. Miller, and with others which their experience might suggest. But mere empirical trials will not answer. The action of any given formula must be tried, not only in the mouth, but in carefully conducted experiments upon artificial cultivations, that results in practical cases may be verified by scientific investigations. We are certain that all such collaboration will be warmly welcomed by Dr. Miller, and we shall be very glad to publish the results in this journal.

